# Migraine in the elderly: clinical characteristics in a series of 71 cases

**DOI:** 10.1186/1129-2377-14-S1-P152

**Published:** 2013-02-21

**Authors:** S Herrero, AL Guerrero, M Ruiz, MI Pedraza, P Mulero, J Barón, I Irene, C De la Cruz, ML Peñas

**Affiliations:** 1 Hospital Clinico Universitario, Spain; 2 Neurology Department, Spain

## Introduction

Though headache prevalence drops markedly in the elderly, this causes a significant burden on the quality of life of this population. However, few studies have investigated primary headaches at this age range.

## Objectives

We aim to analyze characteristics of patients older than 65 attended due to migraine in an outpatient headache clinic in a tertiary hospital.

## Methods

We prospectively considered patients attended from January 2008 to May 2012. Migraine with and without aura was diagnosed accordingly to International Classification of Headache Disorders, II edition (ICHD-II) criteria. For medication overuse headache (MOH) and chronic migraine (CM) we considered ICHD-II revised criteria. We gathered in each patient age, gender, time from onset, complementary tests required, and symptomatic or prophylactic therapies previously used.

## Results

262 patients (189 females, 73 males) out of 1868 (14%) attended in our headache clinic during inclusion period were older than 65 years, and 71 (53 female, 18 male) of them (27.1%) were diagnosed of migraine. Most elderly patients (61, 85.9%) reported that migraine complaints appeared for the first time before 50 years. 26 cases (36.6%) suffered episodic migraine, 18 (25.4%) CM, and 27 (38%) CM with MOH. Only 3 patients (4.2%) were diagnosed of migraine with aura, one of them appearing after 50 years. All patients had received at least one symptomatic treatment, but only 31 (43.6%) had previously used a preventative.

## Conclusion

Migraine represents a burdensome group of elderly patients in our headache clinic. In most of them, onset of migraine was reported before the age of 50. Migraine with aura is infrequent in our series. Chronic migraine is a common diagnosis in our population and medication overuse seems to play an important role. Despite the long time from onset, preventatives were not widely used in our elderly patients before referral.
